# Barriers to the Implementation of Acute Coronary Syndrome Guidelines in Healthcare: A Systematic Review

**DOI:** 10.31083/RCM46736

**Published:** 2026-04-24

**Authors:** Snezana Stolic, Sunitha George, Vainess Mbuzi, Daniel Terry, Xiang-Yu Hou

**Affiliations:** ^1^School of Nursing and Midwifery, University of Southern Queensland, Ipswich, QLD 4350, Australia; ^2^Broken Hill University Department of Rural Health, Susan Wakil School of Nursing and Midwifery, Faculty of Medicine and Health, The University of Sydney, Broken Hill, NSW 2880, Australia

**Keywords:** acute coronary syndrome, barriers, obstacles, clinical practice guidelines, systematic review

## Abstract

**Background:**

Clinical guidelines, pathways, and protocols assist in coordinating care for individuals with suspected or confirmed acute coronary syndrome (ACS). The recently updated 2025 ACS guidelines in Australia and internationally introduced significant changes to improve outcomes compared with the 2016 versions. However, barriers to implementing these ACS clinical guidelines in healthcare settings can lead to significant delays in the management of ACS patients, poor patient outcomes, and increased healthcare costs. This systematic review aims to identify the barriers that healthcare professionals face in implementing ACS clinical guidelines in primary and tertiary healthcare settings.

**Methods:**

Articles were identified through six databases encompassing EBSCO/Cumulative Index to Nursing and Allied Health literature, Cochrane's library, ProQuest, PubMed/Medline, ScienceDirect, and Web of Science, as well as hand searching. The systematic review was conducted in accordance with the Preferred Reporting Items for Systematic Reviews and Meta-Analysis (PRISMA) framework. Search terms included “barriers” OR “obstacles” and “Acute Coronary Syndrome” and “guidelines”. A total of 1625 scientific papers discussing barriers to implementing ACS care guidelines were identified. Following the EndNote automated duplicate process, 67 duplicates were removed. A total of 1558 titles and abstracts were screened using the Joanna Briggs Institute System for Unified Management, Assessment and Review of Information (JBI Sumari). Of the records screened, 1482 were excluded; 76 full papers were retrieved and reviewed. Of these, 68 full-text articles were excluded; 8 studies were included in the final analysis.

**Results:**

The eight included studies were conducted in high-income countries (the USA, Australia, Norway) and low- and middle-income countries (Kenya, Egypt, Indonesia, Sub-Saharan Africa, Tanzania). Sample sizes ranged from 9 to 156,328 participants. Barriers to implementation were grouped into patient-, provider/staff-, and system-level factors. Patient factors included age, gender, race, lack of ACS knowledge, inappropriate healthcare-seeking behavior, delays in treatment, nonadherence to ACS management and lifestyle recommendations, nonattendance at diagnostic tests, language barriers, and geographical distance to the nearest healthcare setting. Provider/staff barriers included deficits in staff knowledge of patient triage, lack of confidence in managing patients with ACS, and deficits in diagnosis and in the provision of recommended management. System factors included inadequate training, the availability of guidelines/protocols, the condition/lack of equipment, and the absence of interventional cardiology units. In addition, factors included reduced available beds, high staff-to-patient ratios, shortages of qualified staff, delays in referral systems, delays in transport and treatment, and openness to adopting new guidelines.

**Conclusion:**

Accurate triage and risk stratification are essential to reduce ACS-related mortality. Health systems should prioritize timely electrocardiogram (ECG) interpretation, transport to percutaneous coronary intervention (PCI)-capable centers, and workforce training. Policy actions must address resource gaps, standardize chest pain pathways, and invest in infrastructure to ensure equitable implementation of the updated 2025 ACS guidelines.

**The PROSPERO Registration:**

CRD42023409325, https://www.crd.york.ac.uk/PROSPERO/view/CRD42023409325.

## 1. Introduction

Acute coronary syndrome (ACS), the clinical manifestation of coronary artery disease (CAD) during an ischemic event, includes ST-elevation myocardial infarction (STEMI), non-ST-elevation myocardial infarction (non-STEMI), and unstable angina [1, 2]. ACS is recognized as the most common reason for patient presentation to emergency departments (EDs) [3]. The underlying pathophysiology of ACS results from acute coronary artery occlusion, predominantly caused by thrombus formation secondary to plaque rupture or coronary spasm [4]. ACS involves impaired reperfusion, which can lead to serious complications such as lethal cardiac arrhythmias, sudden death, cardiogenic shock, or heart failure [5, 6]. These outcomes highlight the clinical urgency of timely diagnosis and interventions.

Although the age-adjusted incidence of CAD is declining due to improved therapies [7], CAD remains the leading cause of mortality worldwide [8], including Australia [9], Europe [10, 11], and the United States of America [9]. Indeed, one in every ten deaths in Australian hospitals is attributed to ACS [12]. In cardiovascular medicine, the management of ACS has long been a critical challenge.

Given the high risk of in-hospital mortality in ACS presentations, accurately identifying ACS and chest pain patients, stratifying risk, and initiating evidence-based pharmacological and interventional management guidelines is critical [13, 14]. The American Heart Association (AHA), American College of Cardiology (ACC), Society of Cardiovascular Angiography and Interventions (SCAI) [15, 16], European Society of Cardiology (ESC), and National Heart Foundation of Australia and Cardiac Society of Australia and New Zealand [17] recommend well-defined guidelines for treating ACS patients.

The 2021 ACC/AHA/SCAI clinical practice guidelines for coronary artery revascularization recommend that people presenting with ACS and chest pain be managed through a structured clinical assessment pathway, supported by anatomical testing, point-of-care, and laboratory-based assays [17]. The management of ACS requires timely in-hospital emergency reperfusion, typically achieved by primary percutaneous coronary angioplasty (pPCI), which may include a drug-eluting stent (DES) or thrombolytic therapy [17]. For NSTEMI, invasive management is generally recommended, although timing may vary based on risk stratification and clinical presentation. In patients with STEMI, thrombolytic therapy remains an option when PCI is not immediately available [6]. The initial management includes antiplatelets, if not contraindicated, alongside pain management using opioids and sublingual nitroglycerin, and continuous cardiac monitoring. Subsequent treatments are based on a STEMI, NSTEMI, or unstable angina diagnosis. The AHA recommends that angiography and pPCI for STEMI be delivered within 90 minutes of presentation to preserve cardiac function [18]. Thrombolytic therapy is recommended if catheterization laboratory facilities are unavailable or patient transfer cannot be achieved within 120 minutes [18]. AHA guidelines also recommend maintaining door-to-needle time for thrombolytic administration to less than 30 minutes to optimize outcomes [18]. However, adherence to ACS guidelines varies, which may be attributed to patient, organizational, and differences in the availability of, and confidence in, the application of protocols and guidelines [1].

Thus, addressing gaps in current management and referral processes and practices is essential to improving ACS care. Therefore, this systematic review aims to identify the barriers faced by healthcare professionals to the implementation of ACS clinical guidelines in primary and tertiary healthcare settings. This review is distinct from previous systematic reviews, as this review evaluates barriers in the context of the newly released 2025 ACS guidelines, which introduce major changes, including updated antiplatelet therapy regimens, revised timing for invasive strategies, and a preference for radial access during PCI. These updates increase implementation complexity across diverse healthcare settings.

## 2. Materials and Methods

This systematic review was conducted using six electronic databases: EBSCO/Cumulative Index to Nursing and Allied Health literature, Cochrane Library, ProQuest, PubMed/Medline, ScienceDirect, and Web of Science to identify original empirical research published between 15 January 2016 and 15 June 2025. This systematic review methodology is based on the 27-item Preferred Reporting Items for Systematic Reviews and Meta-analysis (PRISMA) checklist [19]. The protocol has been registered in PROSPERO (registration number CRD42023409325). Mixed Methods Appraisal Tool (MMAT) version 18 was used to appraise the quality of these studies [20].

To identify articles for inclusion, two reviewers (SS and SG) conducted an anonymized literature search, and an additional reviewer (VM) resolved conflicts. The search strategy incorporated Boolean operators and used Medical Subject Headings (MeSH) and text words in various combinations: (i) Evaluation terms: “barrier*” OR “challenge*” OR “Obstacle*”; (ii) population terms: (“NSTEMI *” OR “STEMI *” OR “Unstable Angina” OR “Acute Coronary Syndrome” AND “patient”); (iii) design terms: (“guideline*” OR “care pathway” OR “protocol”). Exclusion criteria included “review” OR “model” OR “provision of staff education/training interventions” OR “development” OR “risk” OR “prevalence” OR “etiology” OR “criteria” OR “update” (See Appendix A).

The search was limited to “human studies” and those published in English, which introduced language bias. No non-English studies were included due to translation limitations; however, future reviews may consider including these articles to mitigate this language bias. Gray literature was not included in this review; however, a hand search of reference lists identified no additional sources. All potentially relevant articles were imported into the EndNote 21 library for review in accordance with the inclusion and exclusion criteria. The original data search was conducted by authors SG and SS from 22nd of May to 29th of May 2023, and was updated by SS and VM between the 2nd of June and 15th of June 2025.

### 2.1 Study Selection 

**Fig. 1.**
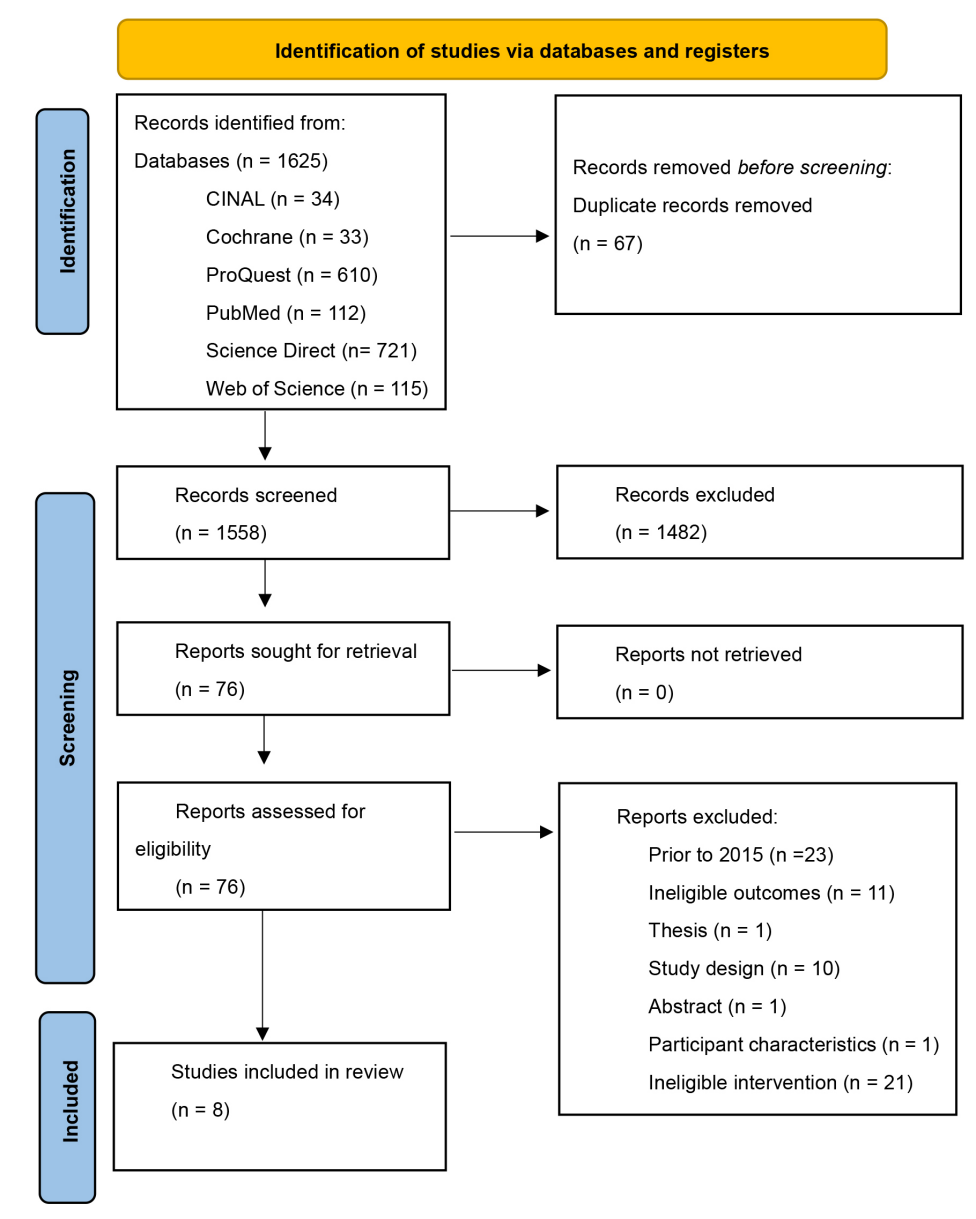
**PRISMA study selection process. Barriers to healthcare professionals in the implementation of ACS guidelines [19]**. PRISMA, Preferred Reporting Items for Systematic Reviews and Meta-Analysis; ACS, acute coronary syndrome.

A total of 1625 articles were retrieved. A total of 67 duplicates were removed manually and using reference management software (EndNote version 21). Of the 1558 extracted papers, the titles and/or abstracts were screened for inclusion and exclusion using the Joanna Briggs Institute for Unified Management, Assessment and Review of Information (JBI Sumari). A total of 1482 articles were excluded, meaning 76 full-text articles remained for eligibility assessment; an additional 68 papers were excluded for various reasons. The final eight studies were used for the systematic review (Fig. 1, Ref. [19] and Table 1, Ref. [1, 21, 22, 23, 24, 25, 26, 27]).

**Table 1.** **MMAT quality appraisal checklist for mixed-methods studies**.

Study	S1	S2	1.1	1.2	1.3	1.4	1.5	3.1	3.2	3.3	3.4	3.5	VM
Bahiru *et al*. [22], 2018	Yes	Yes	Yes	Yes	Yes	Yes	Yes	NA	NA	NA	NA	NA	Yes
Stassen *et al*. [23], 2020	Yes	Yes	Yes	Yes	Yes	Yes	Yes	NA	NA	NA	NA	NA	Yes
Hertz *et al*. [24], 2020	Yes	Yes	Yes	Yes	Yes	Yes	Yes	NA	NA	NA	NA	NA	Yes
Crilly *et al*. [1], 2020	Yes	Yes	Yes	Yes	Yes	Yes	Yes	NA	NA	NA	NA	NA	Yes
Shaheen *et al*. [26], 2021	Yes	Yes	Yes	Yes	Yes	Yes	Yes	NA	NA	NA	NA	NA	Yes
Wihastuti *et al*. [27], 2019	Yes	Yes	Yes	Yes	Yes	Yes	Yes	NA	NA	NA	NA	NA	Yes
Varma *et al*. [21], 2023	Yes	Yes	NA	NA	NA	NA	NA	Yes	Yes	Yes	Yes	Yes	Yes
Bartnes *et al*. [25], 2022	Yes	Yes	NA	NA	NA	NA	NA	Yes	Yes	Yes	Yes	Yes	Yes

S1 = Are there clear research questions? S2 = Do the collected data allow the research questions to be addressed? Qualitative: 1.1 = Is the qualitative approach appropriate to address the research questions? 1.2 = Are the qualitative data collection methods adequate to address the research question? 1.3 = Are the findings adequately derived from the data? 1.4 = Is the interpretation of the results sufficiently substantiated by data? 1.5 = Does coherence exist between qualitative data sources, collection, analysis, and interpretation? Quantitative non-randomized: 3.1 = Is the sampling strategy relevant to address the research question? 3.2 = Is the sample representative of the population under study? 3.3 = Are the measurements appropriate? 3.4 = Is the risk of non-response bias low? 3.5 = Is the statistical analysis appropriate to answer the research question? VM = conflict resolution undertaken by an additional reviewer (VM). Key: yes = criterion is met; no = criterion is not met; NA = not applicable. MMAT, Mixed Methods Appraisal Tool.

### 2.2 Inclusion and Exclusion Criteria

Inclusion criteria were structured using the Population–Concept–Context (PCC) framework to enhance transparency:

∙ Population: People presenting with chest pain and electrocardiogram (ECG) changes or troponin increases, including diagnoses of unstable angina, acute myocardial infarction, NSTEMI, and STEMI, in primary or tertiary healthcare settings.

∙ Concept: Studies examining barriers, challenges, or obstacles to the implementation of ACS clinical guidelines.

∙ Context: Healthcare professionals providing care to the identified population in primary or tertiary care environments.

∙ Study Design: Peer-reviewed quantitative or qualitative research published between 15 January 2016 and 11 June 2025 (reflecting the release of the most recent ACS guidelines by the Cardiac Society of Australia and New Zealand and the American Heart Association).

∙ Language: English only.

In addition, articles that contained any of the following were excluded:

∙ Studies that focused on stable angina, chronic coronary heart disease, or heart failure.

∙ Narrative, integrative, umbrella, or scoping reviews; grey literature; non-peer-reviewed sources; theses; conference presentations; guideline updates; and studies focused on education, development, or model/framework design.

∙ Research addressing etiology, pathophysiology, risk factors, or prevalence of ACS rather than guideline implementation.

### 2.3 Methodological Quality Assessment

Systematic review quality appraisals are used to generate knowledge of different users of reviews. The PRISMA 27-item Checklist, designed primarily to guide the reporting of systematic reviews, was used to guide this review [19]. The studies included in this review were mixed-methods and noncomparative; therefore, the MMAT Quality Appraisal Checklist (see Table 1) was used to assess the quality of these studies [20]. Each MMAT criterion is rated as “yes”, “no”, “cannot tell”, or “not applicable”, where “yes” = criterion is met, “no” = criterion is not met, and “cannot tell” = insufficient or unclear information to determine whether the criterion is met. For example, some studies lacked clarity in data collection procedures or in the justification for sampling strategies, whereas others demonstrated strong coherence between research questions and methods. All eight studies were assessed and deemed high quality, meeting the core criteria for quantitative non-randomized [21, 28] and qualitative designs [1, 22, 23, 24]. However, minor methodological limitations in a few studies could influence the strength of specific findings and should be considered when interpreting the overall conclusions.

### 2.4 Data Extraction and Synthesis

Data were entered into the JBI Sumari for extraction and synthesis by the authors SS and VM, yielding eight papers. The review extracted the first author, dates, geographical location, setting, methods, collected variables, and statistical analysis. The population, the target user of the ACS guidelines, and the outcomes were also included, along with barriers to guideline use (Table 2, Ref. [1, 21, 22, 23, 24, 25, 26, 27]). Data were synthesized using both inductive and deductive thematic approaches. Qualitative and quantitative findings were extracted from each study and organized into categories of barriers to the use of ACS clinical practice guidelines. Quantitative results were translated into qualitative insights through narrative interpretation, thereby enabling integration into a thematic framework. Frameworks such as the Consolidated Framework for Implementation Research and the Theoretical Domains Framework were considered during the design of this review. However, a thematic synthesis approach was retained to allow for greater interpretive flexibility across diverse study designs and to maintain accessibility for a broad clinical and policy audience.

**Table 2.** **Eight articles on implementation barriers by healthcare professionals for ACS guidelines**.

Author/year/geographic location/setting	Methods	Intended or targeted population	Target users	Outcomes
	Variables			
	How statistics or data are analyzed			
Varma *et al*. [21], 2023, USA	**Design**	**Sample and size**	Use of guidelines by HCPs	**Patient-related factors**
**Setting**	Matched case–control study	n = 156,328 patients (total)	To manage patients with NSTEMI	**Age**
USA National Inpatient Sample data of adults with NSTEMI	Cases matched by age and gender NSTEMI, diagnosis, intervention, or a PCI-DES in one artery vs. no intervention for over 80 years compared to under 80 years and outcomes	n = 43,265 aged ≥80 years		∙ ≥80 years were more likely not to receive an intervention (73.3%) vs. those aged <80 years (44.1%)
		n = 113,048 aged <80 years		
				∙ ≥80 years were less likely to receive PCI-DES in one artery (7.7% vs. 18.1%); *p * < 0.0005
				**Type of insurance**
	**Data collection period**			∙ Those with non-Medicare–Medicaid insurance were less likely to receive PCI-DES than those with private insurance
	2016			
	**Variables**			**Gender**
	Healthcare cost and utilization			∙ Less likely to receive PCI-DES if female
				**Race**
				∙ Less likely to receive PCI-DES if non-Caucasian
				**Severity of illness**
				∙ Higher APRDRG risk scores, less likely to receive PCI-DES
				∙ Systems/organization related factors
				∙ Major city hospitals (n = 118) had higher infrastructure scores and expertise scores
				∙ Very remote hospitals (n = 10) had lower scores on infrastructure and expertise
Bartnes *et al*. [25], 2022, Norway	**Design**	**Sample and size**	Use of 2017 European guidelines on diagnosis, management, and transfer of healthcare professionals to manage patients with STEMI	**Patient-related factors**
**Setting**	Retrospective audit of electronic patient records for admissions with AMI	n = 146 AMI patients		**Age**
North Norway Regional Health Authority, including 11 government-run emergency hospitals with acute cardiac care, 1 hospital with 24-hour CL, and 1 hospital with business-hours CL				∙ Multivariable Cox regression identified age differences in receiving reperfusion, with slightly better chances for patients <85 years presenting within 12 hours
	**Data collection period**			
	November 2020 to April 2021			
	**Variables**			**Gender**
	Those requiring acute reperfusion therapy for STEMI			∙ n = 37 (25%) of 146 patients were women
				∙ Women were on average 5 years older
	**Statistics**			**Delays to reperfusion**
	Data entered into Excel and analyzed using Stata software. Shapiro–Wilk W test used to determine prerequisites for parametric testing. Cox regression model used for time-to-event analyses			∙ Transfer from home, 43%, n = 63
				∙ Transfer by ambulance, 38%, n = 55
				∙ Transfer from GP, 11%, n = 16
				∙ Transfer from hospital, 4.1%, n = 6
				∙ Transfer from other, 4.1%, n = 6
	Hazard function assumptions assessed using statistical tests and graphical diagnostics based on Schoenfeld residuals			∙ Median time from CP onset to notification of medical services 72 mins
				**Provider/staffing-related factors**
				∙ Received ECG, 44%, n = 61 out of 137 those who required reperfusion
				∙ Received PHT, 4%, n = 2 out of 48 within 20 mins, median time, 44 mins
				**System/organization-related factors**
				∙ PHT for 33% of individuals, n = 48
				∙ IHT for 14%, n = 20
				∙ pPCI for 47%, n = 68
				∙ CABG for 1% in the acute phase, n = 1
				∙ No reperfusion for 6%, n = 9
				∙ pPCI performed in university hospital 43%, n = 63
				∙ pPCI performed in business hours hospital, 3.4%, n = 5
				∙ pPCI performed in non-CL catchment area, 22%, n = 32 of these 4.8%, n = 7 bypassed their nearest hospital
				∙ Thrombolytic therapy before PCI in the acute or subacute phase, 40%, n = 59
Bahiru *et al*. [22], 2018, Kenya	**Design**	**Sample and size**	Facilitators, context, and barriers to the use of guidelines for ACS care by HCPs	**Patient-related factors**
**Setting**	Qualitative	Snowballing		∙ Delay from symptom onset to hospital presentation
1 hospital: Kenyatta National Hospital	**Data collection period**	16 HCPs		∙ Low levels of knowledge
	Jan–Feb 2017	n = 1 cardiologist,		∙ Inability to afford costs
		n = 2 ED doctor, n = 2 medical officers, n = 3 ED nurses, n = 8 medical registrars		∙ Self-medication
				∙ Language barriers
				**System/organization-related factors**
				∙ Standardized training
				∙ Lack of ECG machines and limited access to diagnostic tests
				∙ Lack of medications
				∙ Lack of policy or hospital guidelines
				∙ Limited beds
				∙ CL available but not for pPCI
				∙ Openness to adopting new quality-of-care interventions
				∙ Staff to patient ratios
Shaheen *et al*. [26], 2021, Egypt	**Design**	**Sample and size**	Current practices, barriers, and areas to improve STEMI management by cardiologists	**Patient-related factors**
**Setting**	Qualitative face-to-face interviews	The number of cardiologists ranged from 1 to 90 depending on the hospital; exact number interviewed not reported		∙ Delays in seeking medical assistance, traffic delays, and delays in locating a pPCI hospital
14 pPCI hospitals	**Data collection period**			
26 non-pPCI hospitals	Jan 2020			∙ Reluctance to undergo procedures
Total: 40 hospitals				**System/organization-related factors**
				∙ Limited EMS role and numbers
				∙ Limited physicians for transfers
				∙ Limited cardiologists to perform pPCI
				∙ Limited nurses for transfers
				∙ No written protocols
				∙ Limited ICU/CCU beds
				∙ Inadequate transfer to pPCI hospitals
				∙ System delays in ED to improve flow
				∙ Inability to transfer to CCU prior to pPCI
				∙ System delays while waiting for CL staff
				∙ Limited equipment, pts required to pay for equipment
				∙ Poor condition of equipment
				∙ Unavailability of therapeutic hypothermia
				∙ Need for standardized training
Wihastuti *et al*. [27], 2019, Indonesia	**Design**	**Sample and size**	Barriers to care of ACS pts by nurses	**Provider/staff-related factors**
	Qualitative survey, using purposive sampling, and semi-structured interviews	n = 16 ED nurses		∙ Differences in confidence between senior and junior nurses
**Setting**				∙ Limitations of the nursing professional role
4 Java hospitals				∙ Unclear job responsibilities
	**Data collection period**			**System/organization-related factors**
	Sep–Dec 2017			∙ Need for standardized training and professional development
				∙ Need for improved collaboration between nursing and physicians
				∙ Lack of peer mentor support programs
Stassen *et al*. [23], 2020	**Design**	**Sample and size**	Barriers and facilitators to implement coronary networks for STEMI pts by HCPs	**Provider/staff-related factors**
Sub-Saharan Africa	Qualitative descriptive	n = 11		∙ Poor recognition of STEMI diagnosis
**Setting**	2 structured in-depth interviews and 2 focus groups	Doctors (n = 5)		∙ Challenges in treatment decisions
Northwest and Northern Cape of Africa		Paramedics (n = 4)		∙ Delays in providing reperfusion
	**Data collection period**	Nurses (n = 2)		**System/organizational-related factors**
	June–Oct 2017			∙ Delays in transport
	**Variables**			∙ CCN under-resourced
	Coronary care network			∙ Unprioritized
	Barriers			∙ Fragmented
	Length of stay			
	IH mortality			
	Readmission with cause related index (1–6 months)			
Hertz *et al*. [24], 2020, Northern Tanzania	**Design**	**Sample and size**	Provider-perceived barriers to diagnosis and treatment of ACS by HCPs	**Patient-related factors**
	Qualitative	n = 11 participants; doctors (n = 6)		∙ Patients lacked knowledge of ACS
**Setting**	**Data collection period**	Clinical officers (n = 5)		∙ Misinterpretation of symptoms
ED and outpatient clinics	2018			∙ Delays in seeking medical assistance
Health centers, community hospital, and 1 referral hospital				∙ Difficulty with self-management
				∙ Non-adherence to medications, follow-up appointments, diagnostic testing, or lifestyle
				**Provider/staff-related factors**
				∙ Poor knowledge
				∙ Inexperience
				**System/organization-related factors**
				∙ Inadequate training regarding ACS
				∙ Lack of diagnostic equipment
				∙ Cost of care
				∙ Referral system delays
				∙ Lack of disease data
				∙ Lack of guidelines
Crilly *et al*. [1], 2020,	**Design**	**Sample and size**	Facilitators and barriers in the ED for rapid CP protocol toward acute coronary syndrome	**Provider/staff-related factors**
Australia	Qualitative	9 clinical staff (n = 4 medical and n = 5 nursing) patients, patients with chest pain		∙ Lack of knowledge
**Setting**	Convenience sampling			∙ Memory, attention, decision processes
1 Royal Brisbane Women’s Hospital ED	Semi-structured interviews			∙ Beliefs about capabilities
	**Data collection period**			∙ Skills/deskilling concern using a pathway
	2016 over 2 weeks			**System/organization-related factors**
				∙ Environmental context and resources

Notes: AHA, American Heart Association; APRDRG, all patient refined diagnosis related grouping; CABG, coronary artery bypass graft; CL, catheterization laboratory; CCN, coronary care network; CP, chest pain; CCU, coronary care unit; ECG, electrocardiogram; ED, emergency department; GRACE, Global Registry of Acute Coronary Events; GP, general practitioner; HCP, healthcare professional; IHT, in-hospital thrombolysis; ICU, intensive care unit; NSTEMI, non-ST-elevation myocardial infarction; outpts, outpatients; PCI, percutaneous coronary intervention; PCI-DES, percutaneous coronary intervention-drug-eluting stent (DES); PHT, pre-hospital thrombolysis; pPCI, primary percutaneous coronary intervention; pt, patient; yrs, years; STEMI, ST-elevation myocardial infarction.

Thematic coding was conducted manually using a structured codebook developed iteratively during the review process. Thematic synthesis followed the steps outlined by Thomas and Harden [29], which include (1) line-by-line coding of findings, (2) grouping codes into descriptive themes, and (3) generating analytical themes. To ensure rigor, two researchers (SS and SG) independently coded the data and discussed discrepancies to reach consensus, supporting inter-coder reliability. Although formal sensitivity or subgroup analyses were not conducted due to the small number of included studies and methodological heterogeneity, heterogeneity was addressed by examining the thematic convergence and divergence across study contexts, populations, and designs. This approach allowed for a nuanced interpretation of recurring barriers while acknowledging contextual variation. Analysis of the eight included studies revealed three recurring factors that were consistently identified and, therefore, extracted as key barriers.

## 3. Results

### 3.1 Characteristics of Studies 

The studies included in this systematic review have different methodologies. Two large studies, one in the United States and the other in Norway, were retrospective audits of electronic databases of non-STEMI and STEMI patients [21, 25]. Six studies were qualitative interviews with cardiologists [26], doctors [1, 22, 23, 24], nurses [1, 22, 23, 27], physician assistants [24], and paramedics [23]. Varma *et al*. [21] investigated disparities in the management of non-STEMI in more than 156,000 patients receiving any percutaneous coronary intervention-drug-eluting stent (PCI-DES) procedure using the 2016 National Inpatient Sample data of adults; meanwhile, Bartnes *et al*. [25] used the 2020–21 North Norway Regional Health Authority of 11 emergency hospitals with a catchment of more than 485,000 residents to investigate barriers against the use of rapid reperfusion in acute STEMI. All conducted qualitative studies were conducted with emergency department participants [1, 22, 23, 24, 26].

Overall, the magnitude of poor adherence to ACS guidelines is high, and this review highlights several key quantitative findings from the included studies. In the United States, a substantially higher proportion of patients aged ≥80 years received no interventional management compared with those <80 years (73.3% vs 44.1%), and this older cohort experienced higher in-hospital mortality rates [21]. Women and non-Caucasian patients were also less likely to receive guideline-recommended interventions, with odds ratios (ORs) of 0.785 and 0.832, respectively [21]. In Norway, only 44% of eligible patients received an ECG, and just 4% received pre-hospital thrombolysis within the recommended 20-minute window [25]. Additionally, 6% of STEMI patients received no reperfusion therapy. In Egypt, 26 of 40 hospitals were unable to perform pPCI, and many reported delays due to staffing and equipment shortages [26]. These figures highlight the prevalence of implementation barriers and underscore the urgency of targeted strategies to improve ACS care delivery (Table 3, Ref. [1, 21, 22, 23, 24, 25, 26, 27]).

**Table 3.** **Study characteristics**.

Author/Year	Country	Setting	Design	Sample	Variables	Target users	Guidelines	Barrier constructs
Varma *et al*. [21], 2023	USA	National Inpatient Sample Data	Matched case–control study	n = 156,328 (≥80 years: 43,265; <80 years: 113,048)	Healthcare cost and utilization	Healthcare professionals	2014 ACC/AHA	Age, insurance type, gender, race, illness severity, hospital infrastructure
Shaheen *et al*. [26], 2021	Egypt	40 hospitals (14 pPCI, 26 non-pPCI)	Qualitative interviews	Cardiologists (number not reported)	STEMI management practices and barriers	Cardiologists	Not specified	Patient delays, EMS limitations, staffing, equipment, protocols
Bartnes *et al*. [25], 2022	Norway	11 emergency hospitals	Retrospective audit	n = 146 AMI patients	Reperfusion therapy needs	Healthcare professionals	2017 ESC	Age, gender, transfer delays, thrombolytic therapy
Bahiru *et al*. [22], 2018	Kenya	Kenyatta National Hospital	Qualitative	n = 16 HCPs	Barriers to ACS care	Healthcare professionals	2013 ACC/AHA	Patient delays, affordability, training, equipment, staffing
Wihastuti *et al*. [27], 2019	Indonesia	4 Java hospitals	Qualitative survey	n = 16 ED nurses	Barriers to ACS care	Nurses	Not specified	Confidence, role clarity, training, collaboration
Stassen *et al*. [23], 2020	Sub-Saharan Africa	Northwest and Northern Cape	Qualitative descriptive	n = 11 (doctors, paramedics, nurses)	Coronary care network barriers	Healthcare professionals	2017 ESC	Diagnosis delays, transport, under-resourced systems
Hertz *et al*. [24], 2020	Tanzania	ED, outpatient clinics, hospitals	Qualitative	n = 11 (doctors, clinical officers)	Barriers to ACS diagnosis and treatment	Healthcare professionals	2018 Tanzania	Knowledge gaps, equipment, cost, referral delays
Crilly *et al*. [1], 2020	Australia	Royal Brisbane Women’s Hospital ED	Qualitative	n = 9 clinical staff	Barriers to rapid chest pain protocol	ED staff	2016 NHF of Aust CSANZ	Knowledge, decision-making, environmental context

ACC, American College of Cardiology; AHA, American Heart Association; ESC, European Society of Cardiology; NHF, National Heart Foundation; CSANZ, Cardiac Society of Australia and New Zealand.

### 3.2 Barriers to Guideline Implementation in Primary or Tertiary Healthcare Settings

**Fig. 2.**
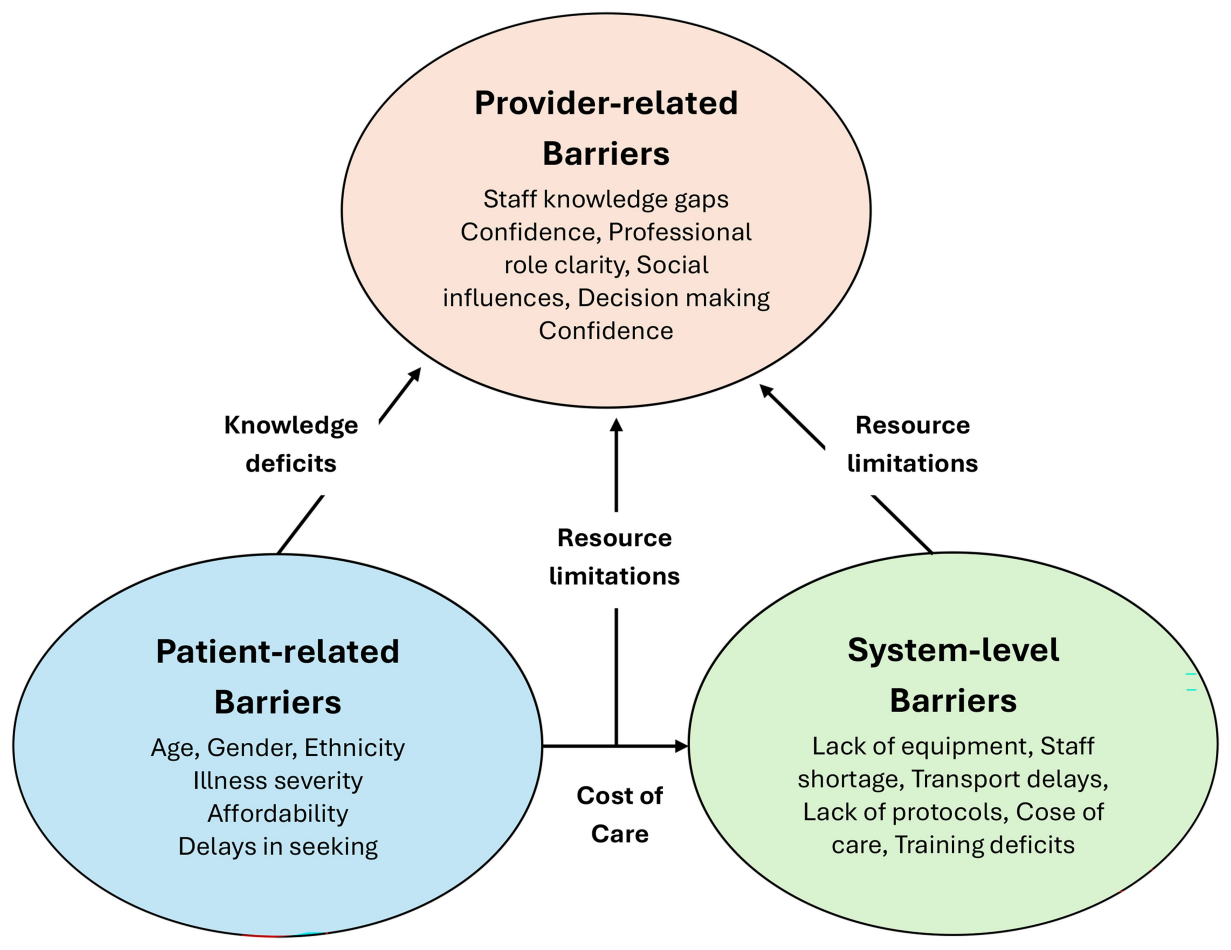
**Conceptual map of the interrelationships between patient-related, provider/staff-related, and system-level barriers to ACS guideline implementation**. Arrows indicate overlapping influences such as knowledge deficits, resource limitations, and cost of care.

While some barriers, such as ‘knowledge deficits,’ are evident across multiple levels, a thematic synthesis approach has been retained to reflect the contextual origin of each barrier, whether patient, provider, or system-related, rather than applying a formal implementation framework. As such, the barriers to implementing ACS guidelines in primary or tertiary healthcare settings were grouped into themes: patient-related factors, provider/staff-related factors, and system/organizational-related factors (Fig. 2).

#### 3.2.1 Patient-Related Factors 

While delays in seeking care are not directly attributable to the adherence of healthcare professionals to ACS guidelines, these delays remain critical public health challenges that affect the timeliness of diagnosis and treatment. Thus, factors highlighted in the literature, such as age, ethnicity, health-seeking behaviors, and access to emergency services, must be recognized as vital elements influencing ACS outcomes. Although these are best addressed through community-level interventions and public education, including these factors in this review reflects their indirect impact on the implementation and effectiveness of guideline-based care.

3.2.1.1 Age Factors

Varma *et al*. [21] found that patients aged 80 years or older were significantly less likely (*p* < 0.0005) to receive any PCI-DES procedure (73.3%) than those younger than 80 years (44.1%). Varma *et al*. [21] found that a PCI-DES procedure for a single artery was associated with improved survival compared with no intervention. Indeed, the mortality rate for those aged under 80 years who received a one-artery PCI-DES was 1.7%, whereas the no-intervention mortality rate for those under the age of 80 years was 4% (*p* < 0.0005). Meanwhile, the mortality rate for those aged over 80 years who received a one-artery PCI-DES was 4.2%, whereas for those aged over 80 years with no intervention, the rate was 9.4% (*p* < 0.0005). Bartnes *et al*. [25] also found that age was a factor; meanwhile, patients aged 85 years and older were less likely to receive reperfusion therapy.

3.2.1.2 Gender, Ethnicity, and Illness Severity Factors

Gender and ethnic disparities were observed in those receiving interventions for patients with NSTEMI. Only 21.5% of women receive reperfusion, and those who do tend to be older [21]. Additionally, women had a lower likelihood of receiving interventions, with an OR of 0.785 (95% confidence interval (CI), 0.766–0.804; *p* < 0.0005), and non-Caucasian patients were also less likely to receive interventions, with an OR of 0.832 (95% CI, 0.809–0.855; *p* < 0.0005) [21]. Patients with a higher all-patient redefined diagnosis-related grouping risk scores were less likely to receive PCI-DES [21].

3.2.1.3 Other Patient Factors

The main reasons patients were delayed in presenting to the hospital and, therefore, represented a barrier to receiving care promptly included: Healthcare seeking behaviors such as delays to presenting to the hospital [21, 22, 24, 26]; treatment adherence and compliance [21, 24, 26], and a deficit in ACS knowledge [21, 22, 23, 24]. These factors were associated with limited patient understanding of ACS symptoms and treatment. Other patient-related reasons included an inability to afford care, self-medication, language barriers, and the broader burden associated with ACS [22, 24]. Regarding insurance status, patients in the United States of America with non-Medicare–Medicaid insurance who were younger than 80 years had a 40% lower likelihood of dying (OR, 0.596; 95% CI, 0.491–0.724; *p* = 0.0005) and a 16% higher chance of not receiving any intervention (OR, 1.160; 95% CI, 1.125–1.197; *p* = 0.0005) [21].

#### 3.2.2 Provider/Staffing-Related Factors

The provider/staffing-related factors included in the results of this systematic review were (i) knowledge, (ii) the professional role, (iii) confidence and beliefs about personal capabilities, (iv) goals, and (v) social influences [22, 23, 27]. Studies have reported a lack of knowledge regarding protocols, guidelines, pathways, diagnostic approaches, and treatment decisions, as well as delays in initiating reperfusion [1, 23, 24]. Crilly *et al*. [1] reported that social professional identity and roles were factors in assessment, management, and referral. Crilly *et al*. [1] also reported that beliefs about staffing capacities and goals influenced protocol implementation, group conformity and support, memory, attention, and decision-making, and that these factors, in turn, affected adherence to guidelines. Another factor was staff-related skills and inexperience [1, 23, 24], with results indicating that hospitals with greater staff expertise performed more coronary angiograms. Interestingly, staffing barriers in high-income settings, such as Australia and Norway, were often linked to role ambiguity, protocol adherence, and professional identity [1, 21, 25]. In contrast, studies from low-resource settings (*e*.*g*., Kenya, Tanzania, Indonesia) emphasized shortages of trained personnel, limited access to continuing education, and lack of standardized training programs [22, 24, 27].

Another staffing factor was the failure to prioritize non-communicable diseases and inefficiencies in patient triage, which demonstrated a clinical knowledge deficit regarding ACS protocols and decision-making tools [23].

#### 3.2.3 System/Organizational-Rated Factors

System/organizational-related factors included in the results of this systematic review were (i) lack of specialized equipment, (ii) lack of hospital beds, (iii) lack of specialist or poor staff skill mix, poor ratio of staff to patients, (iv) cost of care, (v) lack of policy, guidelines and protocols, (vi) transport difficulties, (vii) lack of training programs. Additionally, resource constraints included inadequate equipment (*e*.*g*., ECG machines) and diagnostic tests, as well as a lack of medications (including nitroglycerine and thrombolytics) [1, 22, 23, 24]. Other system-related factors included the lack of standardized protocols, clinical pathways, or hospital guidelines; the absence of a dedicated coronary care unit (CCU) or intensive care unit (ICU) or interventional cardiology; poor equipment conditions [24, 25, 26]. High staff-to-patient ratios [22, 23, 26] and openness to accepting new interventions [22] were also reported.

In low- and middle-income countries (LMICs), system-level barriers were predominantly related to infrastructure gaps, equipment shortages, and limited access to medications [22, 23, 24, 26, 27]. In contrast, system barriers in high-income countries were more often associated with insurance coverage, cost of care, and interdepartmental coordination [21]. Another reason for the presence of barriers to the use of ACS guidelines was reduced pre-hospital thrombolysis use [23, 25], transportation delays, and referral challenges to other healthcare settings [23, 24]. Bahiru *et al*. [22], Hertz *et al*. [24], and Shaheen *et al*. [26] found that the cost of care was a factor in patients not receiving appropriate ACS clinical practice guidelines. Studies have also reported inadequate training and education in the management of ACS, ECG interpretation, and other cardiac emergencies, resulting in fragmented care [1, 22, 23, 24].

### 3.3 Cross-Context Comparison

The studies included in this review span both high-income countries (the USA, Australia, Norway) [1, 21, 25] and LMICs (Kenya, Egypt, Indonesia, Tanzania) [22, 23, 24, 26], offering a diverse perspective on barriers to ACS guideline implementation. While many barriers were consistent across settings, such as knowledge deficits and resource limitations, the underlying causes of these barriers varied. In high-income contexts, barriers were often linked to system inefficiencies, professional role ambiguity, or protocol adherence [1, 21, 25]. In contrast, studies from LMICs highlighted more fundamental structural challenges, including a lack of equipment, limited access to medications, and financial constraints affecting both patients and institutions [22, 23, 24, 27]. Cultural factors, such as health-seeking behaviors and language barriers, were more prevalent in LMICs and influenced timely presentation and treatment adherence [22, 23, 24, 27]. These contextual differences highlight the importance of tailoring implementation strategies to local health system capacities and sociocultural dynamics.

## 4. Discussion

This review critically synthesizes evidence on barriers to implementing ACS treatment guidelines, focusing on patient-, provider/staff-, and system/organizational/-level factors. Thus, by integrating findings from high- and low- to middle-income contexts, this review provides a broader understanding of the structural, economic, and cultural influences on guideline uptake. These insights offer practical implications for tailoring implementation strategies to diverse healthcare settings and inform policy priorities for resource allocation, workforce training, and equity in ACS care delivery.

Implementation of ACS guidelines is challenged by numerous barriers across different settings and contexts; meanwhile, patient characteristics, including age, gender, ethnicity, and geographical location, significantly influence the quality of care delivered [1, 21, 22, 23, 24, 28]. Clinical practice guidelines for patients with ACS recommend that patients receive antithrombotic therapy in the hospital; however, studies have shown that older patients and those with higher body mass index (BMI) are less likely to receive the recommended dose of antithrombotic therapy [30, 31]. Further, older patients and females experience disparities when attending the ED for chest pain and ACS, creating barriers to healthcare professionals adhering to ACS guidelines [31, 32]. Females are more likely to experience pre-hospital ambulance delays [33], less likely to receive an angiogram and revascularization [33, 34, 35], and are less likely to receive an ECG within 10 minutes of arriving at the hospital than men [33, 36].

Indigenous Australians are less likely to receive a coronary angiogram than non-Indigenous Australians, with contributing factors reported as a misdiagnosis of symptoms (16%), substitution with noninvasive procedures (8%), discharge against medical advice (11%), or other unclear reasons (36%) [37]. Geographical location is also another barrier to guideline adherence. People in rural and remote areas of Australia, for example, experience delays due to the distance to the closest health service [38]. A large systematic review indicates that delays in health-seeking behavior among people experiencing symptoms are attributable to limited patient awareness and knowledge [39]. A study of 60 hospitals in China found that the complexity of the condition, lack of recognition of symptoms in people with ACS (including requirements and resources of patients), low government support, movement of staff in ED, management quality, resistance from departments, and overwhelmed staff were barriers to staff knowledge [40]. The engagement of chest pain center staff was associated with knowledge scores; higher scores indicated greater motivation (OR, 1.79; 95% CI, 1.18–2.72), whereas lower knowledge scores indicated implementation barriers (OR, 0.81; 95% CI, 0.67–0.98) [40].

Staffing issues included the need for medical staff training in recognition, triage, and appropriate management of ACS. A study found that patients presenting to the ED had a 5.4-fold higher likelihood of not receiving appropriate triage assessments than those attending an outpatient clinic [7]. Gullick *et al*. [41] compared objective risk assessment using the Australian Global Registry of Acute Coronary Events (GRACE) Risk Tool with standard care for acute coronary syndromes. The study process and evaluation emphasized the implementation of the GRACE risk tool and highlighted the associated impact of the tool on patient management and outcomes. This may indicate that the tool aided clinicians in more accurately predicting risks and making decisions, potentially leading to improved treatment of cardiac presentations. Accurate in-hospital triaging and risk stratification are crucial to avoiding in-hospital death from acute ischemic events. Therefore, the knowledge of nurses regarding symptom identification, ECG interpretation, atypical presentations, and variations is essential in emergency departments and cardiac specialty areas [22, 42]. The model of care employed by nurse practitioners has been highlighted by Davis and Maness [43] as a critical approach to addressing gaps in healthcare facilities, particularly in rural and remote areas. According to guidelines, all patients with chest pain are recommended to receive an ECG within 10 minutes of presentation to the ED. This review reports that this achievement is limited. A study in Ireland using the Acute Coronary Syndrome Application (AcSAP) on Android tablets by experienced triage nurses found a significantly higher proportion of patients who received an ECG within 10 minutes (*p* < 0.001) [44].

Recognizing obstacles and enablers specific to each hospital is crucial for the effective implementation of clinical strategies [45]. In this review system/organization, factors such as the importance of using the chest pain protocol and guidelines are vital. One study has suggested that the use of chest pain pathways may improve patient outcomes. They found that using chest pain protocols resulted in no deaths after 30 days post-discharge from 224 patients [46]. Patients in this same study had a low risk of adverse effects at 12 months post-discharge [46]. ACS pathways should include algorithms and decision-making models [47, 48]; however, not all health organizations adhere to standardized guidelines [49]. Implementation of ACS guidelines necessitates investigation of the persisting barriers. In non-interventional facilities, treatment must be initiated with medical therapy in accordance with guideline recommendations [46]. Other significant deficiencies include shortages of diagnostic equipment and logistical challenges, such as transport delays and fragmented care networks, that impede timely and effective care. In addition, the environmental factors and resource-related barriers in emergency departments further contribute to delayed ACS care [2, 5, 41, 47, 50]. A large systematic review also demonstrated that a lack of pre-hospital care, interfacility care coordination, and interventional cardiology was associated with poorer patient health outcomes [39].

Some facilities are constrained by therapeutic and diagnostic capabilities, resulting in underdiagnosed and undertreated ACS [23, 24, 50]. Across various studies, this review found a lack of essential resources, such as ECG machines, medications, and adequate training, and that organizational guidelines were a common barrier to care. Stassen *et al*. [23] found that resource constraints and transport delays are associated with high mortality among ACS patients. The authors also highlight the need for additional expert policymakers to address the data deficit in disease-burden data, as these gaps hinder effective resource distribution and allocation to affected local facilities. Meanwhile, delays in the referral system have always been a challenge for non-interventional facilities. Indeed, Jacobs *et al*. [51] found that STEMI care implementation was hindered by the absence of triage protocols, reperfusion strategies, interfacility transfer protocols, skilled staff to administer reperfusion therapy, and STEMI plans. These factors can adversely affect care delivery and lead to significant delays in initiating intervention [2, 17]. Healthcare settings must consider the physician-to-nurse staffing ratio, along with bed capacity, when allocating resources to patients undergoing PCI [33].

Overall, these findings highlight the need for targeted implementation strategies that address context-specific barriers. In high-income settings, interventions should focus on workflow optimization, role clarity, and adherence to updated protocols [1, 21, 25, 28]. In low-resource environments, policy priorities include investment in diagnostic infrastructure, workforce training, and financial protection for patients [22, 23, 24, 26, 27]. These insights can inform the design of continuing education and training programs for healthcare professionals that ensure alignment with current ACS guidelines. Digital health tools, such as mobile triage applications and standardized chest pain pathways, could accelerate guideline uptake globally [44, 46]. In addition, these health tools offer scalable solutions for improving ACS diagnosis and management and should be integrated into broader digital ACS care frameworks [49]. Therefore, health system redesign efforts should incorporate these strategies to enhance care coordination, reduce delays, and improve equity in ACS outcomes. Future research should evaluate the effectiveness of these strategies and explore scalable models for improving ACS care equity across health systems [50].

### 4.1 Strengths

A major strength of this review is the inclusion of studies from both high-income countries (USA, Norway, Australia) and LMICs (Tanzania, Kenya, Indonesia, Egypt, North Africa), which provides a broader perspective on barriers to ACS guideline implementation across diverse health systems. Across settings, common themes in patient, provider/staffing, and organizational factors were identified. This diversity enhances the relevance of findings for global policy and practice. To combat the organizational delays, the new USA and ANZ 2025 ACS guidelines both recommend that patients should be transported by emergency medical services rather than private transport and that non-PCI capable hospitals are to be bypassed for PCI capable hospitals to reduce delays to pPCI procedures and to reduce adverse cardiovascular events. Additional strengths include the prospective registration of the review protocol in PROSPERO, adherence to the PRISMA 27-item checklist, and use of the MMAT Quality Appraisal Checklist for mixed-methods studies. A health sciences librarian assisted with the search strategy and initial search, and the review process involved two independent reviewers with conflict resolution procedures, thereby strengthening methodological rigor.

### 4.2 Limitations

Despite these strengths, several limitations should be acknowledged. This review was limited to peer-reviewed empirical evidence on patients presenting with ACS, either by private transport or via emergency medical services, to primary or tertiary healthcare settings. Language restrictions (English-only publications) and exclusion of gray literature may have introduced bias, and the availability of studies from some Asian and European countries was limited, potentially omitting important findings. Heterogeneity across included studies, such as differences in design, outcomes measured, and healthcare contexts, reduced comparability and limits the ability to synthesize findings quantitatively.

Overall, the identified studies had small sample sizes [1, 22, 23, 24] and were single-center [1, 22] or did not include nurses or administrators [24]. Some studies may be subject to selection bias due to retrospective data analysis [21] and observational design, thereby introducing responder bias and representational bias toward larger hospitals [28]. Other concerns were a lack of patient perspectives and involvement of pharmaceutical companies [22]. The predominance of qualitative evidence further limits generalizability and makes cross-context comparisons challenging.

## 5. Conclusion

This systematic review revealed barriers that continue to hinder implementation of ACS guidelines in clinical practice, including the 2025 ACC/AHA/ASPC and the National Heart Foundation and Cardiac Society of Australia and New Zealand Guidelines. Effective healthcare system design and policymaking must prioritize skill maintenance, tailored education, and ongoing updates aligned with new evidence and guidelines. Future research should also incorporate service-delivery trials that directly address these barriers. Future research on service delivery would benefit from trials to address the barriers.

## Data Availability

The datasets from this review are available from the corresponding authors upon reasonable request.

## Abbreviations

ACC, American College of Cardiology; ACS, acute coronary syndrome; AHA, American Heart Association; AKI, acute kidney injury; ARF, acute renal failure; CA, coronary angiogram; CAD, coronary artery diseases; CCL, cardiac catheterization laboratory; CCN, Coronary Care Network; ECG, electrocardiogram; ESC, European Society of Cardiology; MACE, major adverse cardiovascular event; NSTEMI, non-ST-elevation myocardial infarction; PCI, percutaneous coronary intervention; PCI-DES, percutaneous coronary intervention-drug-eluting stent (DES); SD, standard deviation; STEMI, ST-elevation myocardial infarction.

## Supplementary Material

Supplementary material associated with this article can be found, in the online version, at https://doi.org/10.31083/RCM46736.

## Appendix

### Appendix A.

**CINAHL**

((((NSTEMI OR STEMI OR Unstable angina) OR (Acute coronary syndrome)) AND ((Guidelines) OR (Protocol OR Care pathway))) AND (Barriers)) AND ((Patient))

**Cochrane’s Library**

((((NSTEMI OR STEMI OR Unstable angina) OR (Acute coronary syndrome)) AND ((Guidelines) OR (Protocol OR Care pathway))) AND (Barriers)) AND ((Patient))

**ProQuest **

(“non st elevated myocardial infarction”[MeSH Terms] OR (“non st”[All Fields] AND “elevated”[All Fields] AND “myocardial”[All Fields] AND “infarction”[All Fields]) OR “non st elevated myocardial infarction”[All Fields] OR “nstemi”[All Fields] OR “nstemis”[All Fields] OR (“st elevation myocardial infarction”[MeSH Terms] OR (“st”[All Fields] AND “elevation”[All Fields] AND “myocardial”[All Fields] AND “infarction”[All Fields]) OR “st elevation myocardial infarction”[All Fields] OR “stemi”[All Fields] OR “stemis”[All Fields]) OR (“angina, unstable”[MeSH Terms] OR (“angina”[All Fields] AND “unstable”[All Fields]) OR “unstable angina”[All Fields] OR (“unstable”[All Fields] AND “angina”[All Fields])) OR (“acute coronary syndrome”[MeSH Terms] OR (“acute”[All Fields] AND “coronary”[All Fields] AND “syndrome”[All Fields]) OR “acute coronary syndrome”[All Fields])) AND (“guideline”[Publication Type] OR “guidelines as topic”[MeSH Terms] OR “guidelines”[All Fields] OR (“protocol”[All Fields] OR “protocolized”[All Fields] OR “protocols”[All Fields] OR (“critical pathways”[MeSH Terms] OR (“critical”[All Fields] AND “pathways”[All Fields]) OR “critical pathways”[All Fields] OR (“care”[All Fields] AND “pathway”[All Fields]) OR “care pathway”[All Fields]))) AND (“barrier”[All Fields] OR “barriers”[All Fields]) AND (“patients”[MeSH Terms] OR “patients”[All Fields] OR “patient”[All Fields]) AND (clinical pathways) AND (cardiac care) AND (emergency care) AND (emergency response) AND (health disparities) AND (emergency protocols) AND (healthcare access) AND (emergency interventions)

**PubMed/Medline **

((((NSTEMI OR STEMI OR Unstable angina) OR (Acute coronary syndrome)) AND ((Guidelines) OR (Protocol OR Care pathway))) AND (Barriers)) AND ((Patient))

**ScienceDirect**

((((NSTEMI OR STEMI OR Unstable angina) OR (Acute coronary syndrome)) AND ((Guidelines) OR (Protocol OR Care pathway))) AND (Barriers)) AND ((Patient))

**Web of Science**

((((NSTEMI OR STEMI OR Unstable angina) OR (Acute coronary syndrome)) AND ((Guidelines) OR (Protocol OR Care pathway))) AND (Barriers)) AND ((Patient))

**Translations**

NSTEMI: “non-st elevated myocardial infarction”[MeSH Terms] OR (“non-st”[All Fields] AND “elevated”[All Fields] AND “myocardial”[All Fields] AND “infarction”[All Fields]) OR “non-st elevated myocardial infarction”[All Fields] OR “nstemi”[All Fields] OR “nstemis”[All Fields]

STEMI: “st elevation myocardial infarction”[MeSH Terms] OR (“st”[All Fields] AND “elevation”[All Fields] AND “myocardial”[All Fields] AND “infarction”[All Fields]) OR “st elevation myocardial infarction”[All Fields] OR “stemi”[All Fields] OR “stemis”[All Fields]

Unstable angina: “angina, unstable”[MeSH Terms] OR (“angina”[All Fields] AND “unstable”[All Fields]) OR “unstable angina”[All Fields] OR (“unstable”[All Fields] AND “angina”[All Fields])

Acute coronary syndrome: “acute coronary syndrome”[MeSH Terms] OR (“acute”[All Fields] AND “coronary”[All Fields] AND “syndrome”[All Fields]) OR “acute coronary syndrome”[All Fields]

Guidelines: “guideline”[Publication Type] OR “guidelines as topic”[MeSH Terms] OR “guidelines”[All Fields]

Protocol: “protocol”[All Fields] OR “protocol’s”[All Fields] OR “protocolized”[All Fields] OR “protocols”[All Fields]

Care pathway: “critical pathways”[MeSH Terms] OR (“critical”[All Fields] AND “pathways”[All Fields]) OR “critical pathways”[All Fields]
